# Follow-Up Magnetic Resonance Imaging of Sagittal Groove Disease of the Equine Proximal Phalanx Using a Classification System in 29 Non-Racing Sports Horses

**DOI:** 10.3390/ani14010034

**Published:** 2023-12-21

**Authors:** Josephine E. Faulkner, Zoë Joostens, Bart J. G. Broeckx, Stijn Hauspie, Tom Mariën, Katrien Vanderperren

**Affiliations:** 1 Department of Morphology, Imaging, Orthopaedics, Rehabilitation, and Nutrition, Faculty of Veterinary Medicine, Ghent University, Salisburylaan 133, 9820 Merelbeke, Belgium; 2 Equitom Equine Clinic, Paalstraat 8, 3560 Lummen, Belgium; 3 Department of Veterinary and Biosciences, Faculty of Veterinary Medicine, Ghent University, Heidestraat 19, 9820 Merelbeke, Belgium

**Keywords:** P1, fissure, subchondral bone, bone stress, bone fatigue, MRI, horse

## Abstract

**Simple Summary:**

Sagittal groove disease (SGD) of the equine proximal phalanx is considered to be a chronic bone stress injury and reported to have variable evolution. The aim of this study was to describe SGD on sequential low-field magnetic resonance imaging (MRI) examinations using a classification system. The 29 horses included were predominantly warmbloods used for show jumping and had repeat MRI examinations ranging from 1 month to 3.2 years from initial examination. During the initial rehabilitation period, evolution of SGD MRI classification varied among horses. Importantly, two limbs with a subchondral microfissure and one with subchondral demineralisation progressed to incomplete macrofissure/fracture, indicating that these classifications should be considered a potential prodromal sign of macrofissure formation and horses with these findings should be carefully managed to minimise the risk of catastrophic fracture propagation. In this study, successful return to full training and competition was achieved in 86% of horses with SGD; however, 20% of horses re-presented for MRI with recurrent lameness after varying time periods. Further research is needed into patterns of progression of SGD on a larger scale with the objectives of improving risk assessments, prognostication and treatment plans.

**Abstract:**

Evolution of magnetic resonance imaging (MRI) findings in horses with sagittal groove disease (SGD) of the proximal phalanx is relatively sparsely described. This retrospective, descriptive, longitudinal study describes the findings of sequential low-field MRI fetlock examinations in horses with SGD of the proximal phalanx using a classification system. Twenty-nine horses were included, predominantly warmbloods used for show jumping (79%). For 29 limbs re-examined during the initial rehabilitation period, classification remained constant (n = 18), increased (n = 2), decreased (n = 7), and fluctuated (n = 2). Notably, two limbs with initial classification 4b (bone oedema-like signal with subchondral microfissure) and one with 4c (bone oedema-like signal with subchondral demineralisation) progressed to classification 5 (incomplete macrofissure/fracture), highlighting their potential as prodromal or imminent fissure pathology. Following conservative (n = 28) and surgical (n = 1) treatment, 86% of the horses re-entered full training and competition with a mean ± sd recovery time of 9.4 ± 4.4 months. In total, 20% of horses in the study subsequently presented for repeat MRI due to recurrent lameness after resuming full work, with classification that was the same (n = 2), increased (n = 2), or decreased (n = 2) compared with the last scan. This study underscores the variability in progression of SGD MRI findings, emphasising the need for further larger-scale research into patterns of progression.

## 1. Introduction

Sagittal groove disease (SGD) of the proximal phalanx (PP) of equine athletes is predominantly considered to be a maladaptive response to chronic bone stress overload and may be associated with acute, chronic and/or intermittent lameness [1,2,3,4,5,6,7,8,9,10,11,12,13].

Reported pathologies that can be detected with diagnostic imaging include subchondral osseous densification (also referred to as sclerosis), subchondral bone demineralisation (also referred to as resorption or osteolysis), bone oedema-like signal (also referred to as bone oedema-like lesions, bone marrow lesions or fluid signal), osseous cyst-like lesions, fissures and incomplete and complete fractures [1,2,3,4,5,6,7,10,11,12,13,14,15,16,17]. Magnetic resonance imaging (MRI) is regarded as the gold standard diagnostic imaging modality for diagnosis of SGD due to its ability to detect stress injuries in earlier phases than other modalities [2,3,4,5,6,7,11,12,18]. The small number of case series that describe follow-up MRI findings in SGD have shown a considerable variation in evolution with time, ranging from improvement (e.g., resolution or decreased severity of bone oedema-like signal and healing of incomplete fractures) to unchanged findings (e.g., persistent bone oedema-like signal) and deterioration (e.g., increased subchondral bone loss and development of osseous cyst-like lesions) [2,5,7]. Catastrophic propagation of fissures and incomplete fractures in the sagittal groove (SG) are reported, but it is currently unknown whether other forms of subchondral trauma in this region are at risk of progressing to fracture [5,13]. To date, follow-up of MRI imaging findings over time periods exceeding 10 months are not reported.

Clinical outcomes following conservative and surgical treatment of SGD are described, with successful and unsuccessful performance outcomes described for both methods [2,3,4,5,7,13,15,16,17,19,20,21,22]. It has been suggested that different injury types in the SG may be associated with different outcomes [2]. In human medicine, an MRI classification system for stress injuries of the tibia, the Modified Fredericson MRI Classification System, has been validated for use in predicting time to return to sports activity [23,24]. This classification is based on pathological progression of tibial stress injuries, and categorisation on MRI is dependent on the presence of periosteal oedema, bone marrow oedema and intracortical signal abnormality. A classification system reflecting the suspected pathological progression of bone stress injuries in the SG in horses has not yet been applied to clinical cases in a longitudinal fashion. If patterns are found in lesion progression, the classification system may be used to assist with risk assessment, prognostication and formulation of rehabilitation programs.

It was hypothesised that the MRI findings associated with bone stress injury in SGD may improve, remain static or worsen during the rehabilitation period following initial diagnosis. The aim of this study was to describe the time-related progression of MRI findings in SGD using a classification system formulated to represent suspected pathological progression of chronic overload/bone stress injury.

## 2. Materials and Methods

### 2.1. Case Selection

The study was retrospective, descriptive and longitudinal in design. No approval by an ethical committee was required. Inclusion criteria were horses greater than 1 year old that underwent low-field MRI examination of a fetlock or multiple fetlocks in a standing position and were diagnosed with abnormalities of the SG of the PP, with at least one follow-up MRI examination of the affected fetlock/s after the initial MRI diagnosis. MRI examinations were performed at Equitom Equine Clinic between March 2014 and June 2023. The horses were identified from a group of 111 horses (132 limbs) with SGD that were included in another study evaluating the findings at the initial MRI examination (age range 1.3–18.5 years). Follow-up MRIs had been performed on the request of referring veterinarians, taking into consideration advice from the radiologist and with owner consent.

Cases were found by keyword search of archived reports by one author (European College of Veterinary Diagnostic Imaging (ECVDI) certified radiologist, Radiologist 1). Clinical records were collected and anonymised by the same author. The original MRI reports were written predominantly by ECVDI-certified radiologists (2016–2023) with a small number written by an experienced and competent diagnostic imaging veterinarian (2014–2018).

Lame horses that underwent multi-limb MRI and had SGD changes described in all examined limbs were assumed to have lameness in all limbs for the purpose of the study even if it was not explicitly stated in the clinical history. Levels of rehabilitation and exercise were grouped as rest (box, paddock, hand/ridden walking), base level rehabilitation (flat work, cavaletti and basic movements only), advanced rehabilitation (jumping, advanced movements), and full training ± competition (considered recovered). Decisions on management were made by the referring veterinarian and owner, with consideration given to the recommendations of the radiologist. Information on training and competition performance levels was sourced from clinical records and publicly available competition results websites (www.FEI.org (accessed 8 June 2023) and www.hippomundo.com (accessed 8 June 2023)) by Radiologist 1.

### 2.2. MRI Examination Protocol

All cases underwent examination of the fetlock with an open 0.27T permanent magnetic resonance system (Hallmarq EQ2, Guildford, UK) under sedation. Both forelimbs/hindlimbs were not routinely examined unless this was clinically indicated and agreed to by the referring veterinarian and/or owner. Standard/typical MRI acquisition protocols are presented in Supplementary File S1. MRI studies were retrieved and anonymised by Radiologist 1. The initial MRI examination (T = 0) and those acquired during the following rehabilitation program were grouped as occurring in the “initial period”. MRI examinations completed following a period back in full work were grouped as “subsequent periods”.

### 2.3. MRI Evaluation

Images were retrospectively graded by an ECVDI-certified radiologist (Radiologist 2) who was blinded to the horse signalment and clinical history. Cases were evaluated using a DICOM viewer (Horos v.4.0.0 RC5, Annapolis, MD USA, horosproject.org) in a random order (Random Sequence Generator: www.random.org/sequences (accessed 6 February 2023)). Sequential MRI examinations for each limb were assessed in date order, and the assessor was aware that they were examining images of the same limb over time.

The image assessment was performed using all MRI sequences available for each fetlock examination. Semi-quantitative grading schemes were adapted from previous publications and subchondral bone defects, subchondral demineralisation, osseous densification and bone oedema-like signal were graded as described in Supplementary File S2 [1,16,18,25]. The SG was divided into three subregions: dorsal, middle and palmar/plantar thirds. For each category, the highest ranked grade from the three subregions was assigned as the overall grade for that limb. If it was impossible to evaluate a criterion due to a lack of appropriate sequences or excessive artefact, this was noted as “cannot evaluate”. Examinations with insufficient sequences for adequate assessment of the majority of criteria were excluded on agreement by Radiologists 1 and 2.

Following grading, the SG was scored according to an SGD MRI classification system that was based on the concepts of the Modified Fredericson MRI Classification System to reflect suspected pathways of pathological progression and severity of chronic overload/bone stress injury, with adaptations made for the articular nature of SGD in horses (Table 1).

In the final stage of image assessment, Radiologist 2 cross-checked the grading and classification data with the original MRI reports. In the case of a discrepancy in the severity of a lesion, the grade according to the grading scheme took precedence over the term used in the original report. In the case of a discrepancy regarding the presence or absence of a lesion in the SG, the images were re-evaluated by a second blinded EVCDI-certified radiologist (Radiologist 3) who made the deciding verdict (majority rule decision-making).

### 2.4. Data Analysis

The raw score data for the semi-quantitative grading scheme and SGD MRI classification system were presented as ordinal variables. Progression of classification over time was categorically grouped (increased, static, decreased or fluctuating). Level of rehabilitation and exercise were collected as categorical data. Time between MRI examinations and time to return to full work were collected as continuous data. Level of performance reached once in full work was categorically grouped (same or higher level, lower level, unknown level). Data were collected using Microsoft Excel (Version 16.72, Microsoft Corporation), and descriptive analysis was performed using the same program.

## 3. Results

### 3.1. Horses

**Figure 1:**
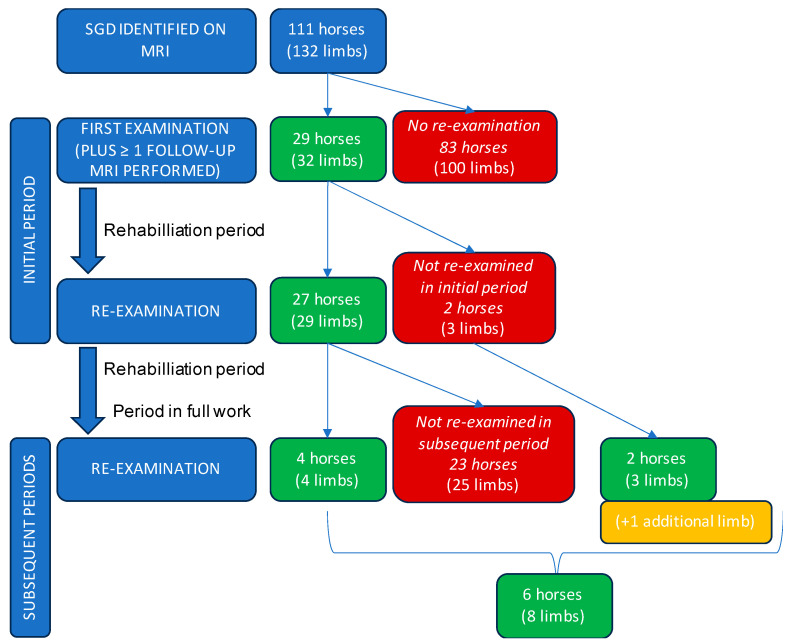
Flow chart overview of the number of horses and limbs with sagittal groove disease (SGD) included in the study, with green and orange boxes representing those included in the initial and subsequent periods.

A total of 29 horses with SGD that underwent two or more repeated fetlock MRI examinations were included in the study (16 mares, 2 stallions, 11 geldings). Mean ± sd age at initial MRI diagnosis was 9.7 ± 2.3 years (range 6–14.9 years). Breeds included 28 warmbloods and one pony. Discipline of the horses included 23 show jumping (FEI 5* (n = 4), 4* (n = 3), 3* (n = 10), 2* (n = 1), 1* (n = 1), national level, e.g., Young Horse Championship (n = 4)), 5 dressage (FEI CDI 5* (n = 2), 3* (n = 2), national level (n = 1)) and 1 eventing (FEI 4*). Twenty-seven horses were in full training and competition until shortly prior to the MRI referral. Two horses were already being rested at the time of initial MRI (2 and 3 months’ duration, respectively). An overview of clinical information at the first MRI examination and during the initial rehabilitation period is shown in Table 2. A total of 29 horses (32 limbs) were re-examined during the initial period. A total of six horses (8 limbs) subsequently re-presented for MRI examination at later dates due to recurrence of lameness after variable periods in full work. A flow chart overview of the number of horses and limbs included in the study from the base sample population of horses identified with SGD is shown in Figure 1.

### 3.2. MRI Acquisition

Fetlock MRI protocols varied between examinations, as shown in Supplementary File S1. The majority of horses underwent sequential MRI scans in one limb with SGD (n = 25), whereas the remaining four horses had MRI scans of both forelimbs. In total, 33 limbs with sequential examinations were included in the study: 12 left forelimb (LF), 14 right forelimb (RF), 2 left hindlimb (LH), 5 right hindlimb (RH). Five horses had an additional limb diagnosed with SGD on MRI but without sequential scans acquired; one was scanned at the time of initial MRI examination and four at the time of a follow-up examination. These limbs were excluded from the study.

Most limbs underwent two sequential scans (n = 24), and the remainder underwent three scans (n = 4), four scans (n = 1), five scans (n = 2) and six scans (n = 2). Two of the horses with scans of both forelimbs underwent two examinations of each limb at the same time points (n = 2). One horse had both forelimbs scanned in the initial three examinations but only one in the subsequent occasions (LF, n = 6; RF, n = 3). The other underwent three scans of one forelimb before having the contralateral scanned (RF, n = 4; LF, n = 2).

### 3.3. MRI Evaluation

On comparison of the blinded grading results with the original reports, there were 16 discrepancies concerning identification of lesions: subchondral defect (n = 7), subchondral demineralisation (n = 2) and bone oedema-like signal (n = 7). Radiologist 3 agreed with the assessment of Radiologist 2 in nine cases and agreed with the original report in seven cases. The grades were altered accordingly before data analysis.

### 3.4. Initial Period

#### 3.4.1. SGD MRI Classification

**Figure 2:**
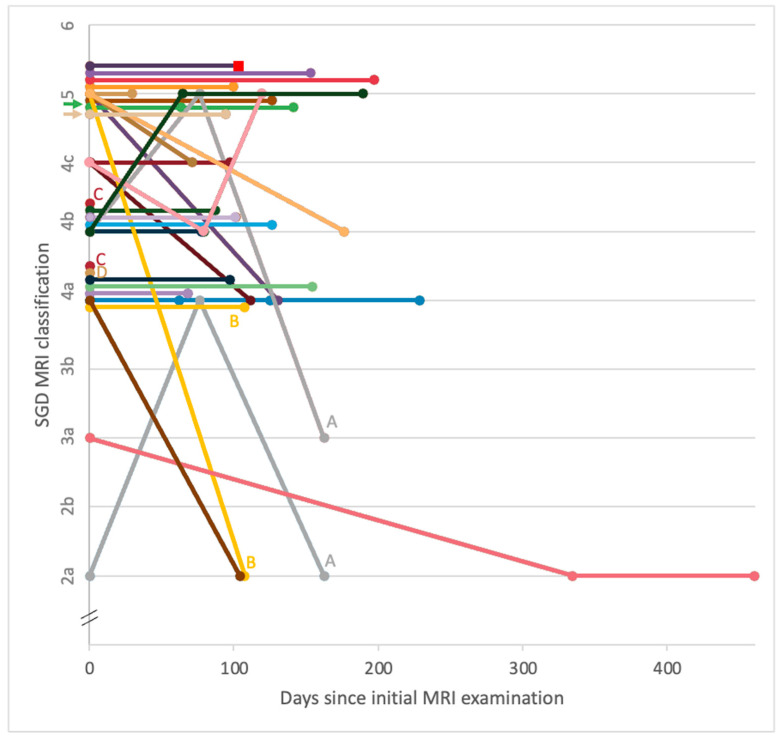
Line graph showing evolution of sagittal groove disease (SGD) MRI classification from initial MRI examination for 29 horses with SGD (32 limbs) during the initial rehabilitation period. Overall, throughout the initial period, eighteen limbs maintained the same classification, two increased, seven decreased and two fluctuated. Three limbs were not re-examined in the initial period. *Notes. Each plotted circle represents the results of one MRI examination, with different colours representing different horses (colours consistent across both charts). Lines join the sequential examinations for each limb (initial examination, t = 0). Lettered labels A–D indicate bilateral forelimbs of the same horse. Coloured arrows on the y-axis indicate limbs of horses that were at rest for 2–3 months prior to the initial MRI examination; the remainder of horses were in full work until more recently. Red squares indicate the timing of an MRI examination just before CT-guided surgical treatment was pursued*.

At initial presentation, SGD MRI classifications were 2a (n = 1), 3a (n = 1), 4a (n = 8), 4b (n = 7), 4c (n = 3) and 5 (n = 12). No limbs had an overall classification of 0, 1a, 1b, 2b, 3b or 6. In three horses with bilateral forelimb SGD detected at the time of the initial MRI examination, there was a different classification in each forelimb (4b/2a, 4a/5 and 4a/4b, respectively). The evolution of SGD MRI classification during the initial rehabilitation periods for each limb is shown in Figure 2.

Limbs with repeat MRI examinations in the first 3 months (n = 11) had an MRI SGD classification that had remained static (n = 6, 55%), increased (n = 3, 27%) or decreased (n = 2, 18%). Limbs re-examined between 3 and 6 months (n = 22) had classification that was stable (n = 14, 64%), increased (n = 1, 5%) or decreased (n = 6, 27%) relative to the initial scan. In scans acquired between 6 and 9 months (n = 3), all had the same MRI SGD classification as the first scan (n = 3, 100%). For the one limb scanned between 9 and 12 months and 15 and 18 months, it maintained a lower MRI SGD classification compared with the first scan on each occasion. Overall, for the 29 limbs re-examined during the initial rehabilitation period, 18 limbs maintained the same classification during all scans performed, while 11 had a change in classification (increased (n = 2), decreased (n = 7), fluctuating (n = 2)).

#### 3.4.2. Rehabilitation and Performance

All 29 horses were initially managed conservatively with individually tailored rehabilitation programs. One horse (initial classification 5) underwent CT-guided cortical screw fixation of the PP after 3 months of rehabilitation due to persisting lameness.

In total, 25 horses were identified to have re-entered full training/competition. Of these, timing was available for 18 horses with a mean ± sd [range] time taken to resume full training and competition of 9.4 ± 4.4 [5,6,7,8,9,10,11,12,13,14,15,16,17,18,19,20] months, and timing was not available for seven horses. Three horses were lost to follow-up, and one was in ongoing rehabilitation at the time of data collection. In the absence of validation of the classification system with inter- and intra-observer studies and also due to small group sizes, comparisons of the time taken to re-enter full work for the SGD MRI classification groups are not presented.

Twenty-four horses recovered with conservative treatment to re-enter full training and competition. Of these, seventeen performed to the same or a higher level to pre-injury levels (initial classifications 4a (n = 6), 4b (n = 3), 4c (n = 1), 5 (n = 7)) and three horses that recovered performed at a lower level than previously (initial classifications 4c (n = 2), bilateral 4a/4a (n = 1)). Four additional horses were reported to have resumed full work but performed to an unknown level (initial classifications 4b (n = 2), 5 (n = 1), bilateral 4a/4b (n = 1)). The horse that underwent surgical treatment reached full performance at 11 months post-surgery.

### 3.5. Subsequent Periods

**Figure 3:**
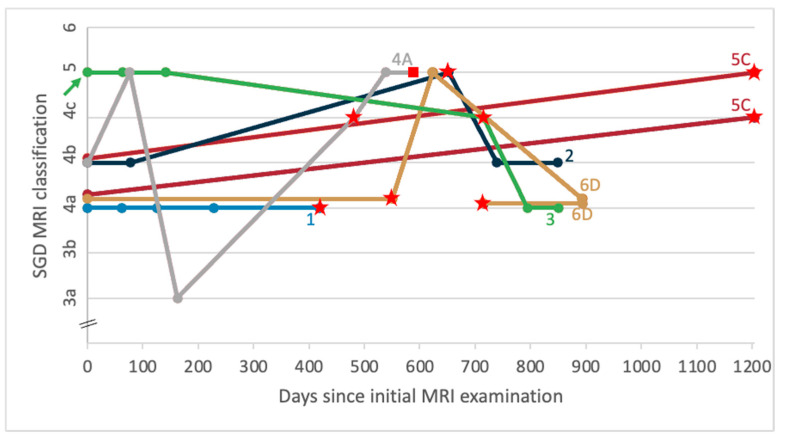
Line graph showing evolution of sagittal groove disease (SGD) MRI classification from initial MRI examination for 6 horses (8 limbs) that re-presented for MRI after recurrence of lameness following a period in full work. *Notes. Each plotted circle represents the results of one MRI examination, with different colours representing different horses (colours consistent across both charts). Lines join the sequential examinations for each limb (initial examination, t = 0). Numbered labels 1–6 indicate horses as referred to in* Table 3*. Lettered labels A, C, D indicate horses with bilateral forelimbs scanned. Red stars indicate a repeat MRI examination due to recurrence of lameness following a period in full training and competition. A coloured arrow on the y-axis indicates a limb in a horse that was at rest for 2–3 months prior to the initial MRI examination; the remainder of horses were in full work until more recently. Red squares indicate the timing of an MRI examination just before CT-guided surgical treatment was pursued*.

Following a period back in full work, six horses were referred for repeat MRI by veterinarians due to recurrence of lameness. The evolution of SGD MRI classification for these horses is shown in Figure 3. Duration of rehabilitation periods and time to reach full performance for each horse are shown in Table 3. Time taken for these horses to resume full training and competition during the first rehabilitation period had ranged from 6 to 15 months. The horses re-presented for MRI due to recurrent lameness between 3 and 15 months after restarting full work. Time taken to resume full work in the second rehabilitation period ranged from 6 to 14 months. One horse (Horse 6) re-presented for MRI due to recurrent lameness for a second time. This horse had spent 6 months in the second rehabilitation period and had been in full work for 2 months.

## 4. Discussion

About one-quarter of the population of horses diagnosed with SGD on MRI at Equitom Equine Clinic (29/111 horses) underwent follow-up MRI. Repeat MRI was not performed in all cases, and this decision is likely to have been influenced by the clinical history, clinical findings, preferences of the referring veterinarian and owner, financial considerations, the advice of the radiologist/diagnostic imaging veterinarian and/or the treatment pursued.

Initial MRI examinations in this study included classifications 2a (mild osseous densification), 3a (subchondral microfissure with mild osseous densification), 4a (bone oedema-like signal), 4b (bone oedema-like signal with subchondral microfissure), 4c (bone oedema-like signal with subchondral demineralisation) and 5 (incomplete macrofissure/fracture). Absence of classifications 1a (minor subchondral defect), 1b (microfissure < 3 mm proximodistal length), 2b (moderate to severe osseous densification), 3b (subchondral microfissure with moderate to severe osseous densification) and 6 (complete fracture) originate from low or absent prevalence identified within the sample population. The relative low frequency of classifications 2a and 3a may emanate from selection bias, as these findings are considered to be of minor clinical relevance, therefore recheck examination is less likely to be advised and subsequent lameness is less likely to occur. The horses with these classifications had contralateral SGD of increased severity (n = 1) and concurrent marked osseous densification and bone oedema-like signal in the metacarpal condyle (n = 1), findings which were considered more clinically relevant.

Similar to previous publications, the majority of the follow-up scans occurred in the first 6 months of rehabilitation [2,5,7]. Most scans during this interval were performed on horses in the rest period, a smaller number were in the base phase of the rehabilitation program and only one horse was in the advanced phase of rehabilitation. Previous publications have reported MRI findings up to a maximum duration of 10 months after the initial scan [2,5,7]. In this study, seven horses had repeat MRI examinations acquired more than one year after the first MRI; one horse was still in rehabilitation and six horses had re-entered full work and had recurrence of lameness.

**Figure 4:**
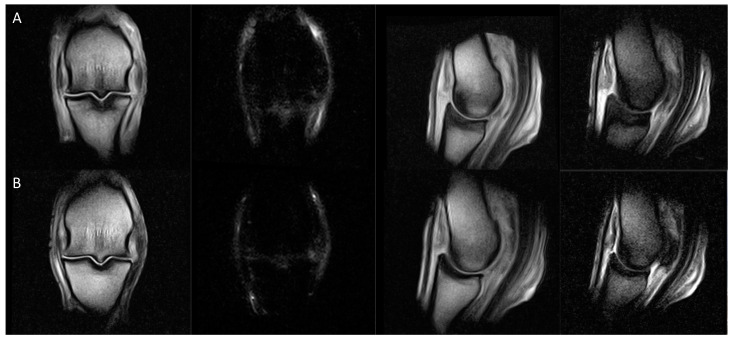
From left, dorsal T1W GRE, dorsal STIR FSE, sagittal T1W GRE and sagittal T2 GRE images of the left hindlimb fetlock of a horse with decreased sagittal groove disease (SGD) MRI classification between (**A**) the initial examination (classification 5) and (**B**) repeat examination 6 months later, at the end of the rest period of the rehabilitation program (classification 4b). The extent of bone oedema-like signal associated with the sagittal groove has markedly reduced, and a faint linear hyperintense microfissure persists in the subchondral bone plate but is no longer extending into the trabecular bone. There is concurrent osteophytosis of the fetlock joint and densification and bone oedema-like signal in the dorsal aspect of the sagittal ridge of the third metatarsus. Notes. T1 GRE, T1-weighed gradient recalled echo; STIR FSE, short tau inversion recovery fast spin echo; T2 GRE, T2-weighted out of phase gradient recalled echo (T2*oW).

Follow-up MRI in the initial period showed variable evolution of the findings in the SG, which is similar to previous publications [2,5,7]. The same SGD classification was maintained throughout all examinations performed in the initial period in 62% of limbs. Decreased classification compared with the initial scan was seen in 18% of limbs examined between 0 and 3 months and in 27% of limbs examined between 3 and 6 months, with evidence of healing of subchondral defects and demineralised areas and/or resolution of bone oedema-like signal (Figure 4). No clear pattern was apparent in the rate of decrease or subsequently attained classification. For example, four fetlocks with initial classification 5 decreased to 2a, 4a, 4b and 4c during the 6 months after the initial MRI examination.

**Figure 5:**
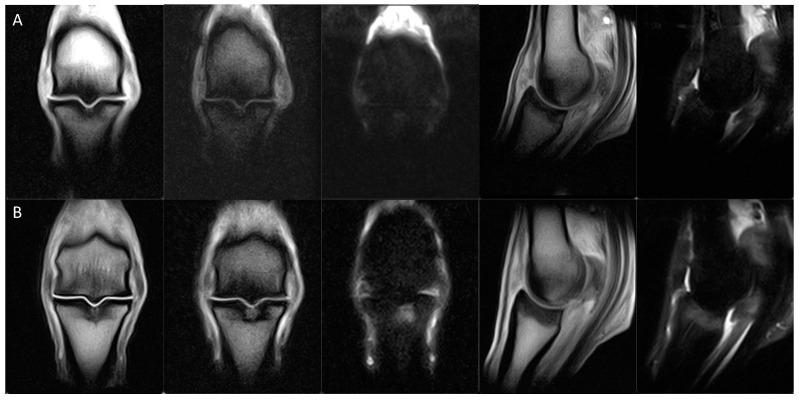
From left, dorsal T1W GRE, dorsal T2 GRE, dorsal STIR, sagittal T1W GRE and sagittal STIR images of the left forelimb fetlock of a horse with increased SGD MRI classification between (**A**) repeat presentation for MRI following a period in full work (classification 4c) and (**B**) 3.5 months later during the rest period of the rehabilitation program (classification 5). There is an increase in bone oedema-like signal and development of a visible proximodistally oriented tripartite macrofissure, which is associated with the previously identified demineralisations in the subchondral bone plate of the SG. There is concurrent osteophytosis of the fetlock joint and densification of the dorsal aspect of the sagittal ridge of the third metacarpus. Notes. T1 GRE, T1-weighed gradient recalled echo; T2 GRE, T2-weighted out of phase gradient recalled echo; STIR FSE, short tau inversion recovery fast spin echo (T2*oW).

A proportion of limbs increased in classification at follow-up scans, highlighting that the severity of pathology on initial MRI examination may be underestimated. In the period between 0 and 3 months, 27% of limbs examined had an increased SGD classification. During this period, one limb had an initial classification of 2a and subsequent classification of 4a. The authors propose that this apparent development of increased bone oedema-like signal may be due to physiological decrease in osseous densification during rest, unmasking previously obscured mild bone oedema-like signal. Development of hyperintense STIR signal in the metacarpal condyle or more specifically in the dorsodistal aspect of the sagittal ridge has been described in horses after 3 months of rest, which is likely compatible with a similar process [5,26]. In steeplechasers the hyperintense STIR signal was accompanied by a decrease in bone density as measured on CT using Hounsfield Units [26]. Alternatively, increased bone oedema-like signal in the SG may represent oedema or another process, for example, hyperaemia, haemorrhage, inflammation, fibrosis or necrosis [27]. In two limbs with classification 4b, the classification increased to 5. Possible explanations include increased conspicuity of an occult macrofissure secondary to resorption at the fissure margins, development of macrofissure in a region of existing osseous microtrauma and/or distal propagation of an existing subchondral microfissure. One limb, which decreased from classification 4c to 4b during the first 3 months of a rehabilitation period, subsequently increased to classification 5 between 3 and 6 months while the horse was in the base level of rehabilitation (Figure 5). Although a fissure did not become apparent in all cases with classification 4b and 4c, these classifications should be considered potential prodromal or imminent fissure/fracture pathology. Further investigation is required to determine whether withdrawal from exercise and treatment curtails the development of visible fissures in some cases or whether certain MRI features alter the likelihood of macrofissure development. Additionally, the reasons for the slow rate of healing of sagittal fissures compared with fissures in other regions needs to be investigated. In some surgically treated cases, it is reported that radiographic healing is not observed even after a long period, and in others, healing is reported to occur slowest at the articular aspect, therefore synovial communication may play a role [15,19]. Alternatively, it may be due to the presence of continued stress and strain in the SG, which may be present even in a horse in a rest phase of rehabilitation, for example, during weightbearing while standing or walking [9,28,29,30,31]. Tight turns in the stable or while being walked out could also play a role [32,33]. Due to the variability in progression of SGD shown in this study, it is likely that follow-up MRI examinations are important for establishing how severe the injury is and guiding further management. Further investigation with a higher frequency of repeat MRI examinations performed at more regular intervals in a larger numbers of horses will be required to confirm this.

**Figure 6:**
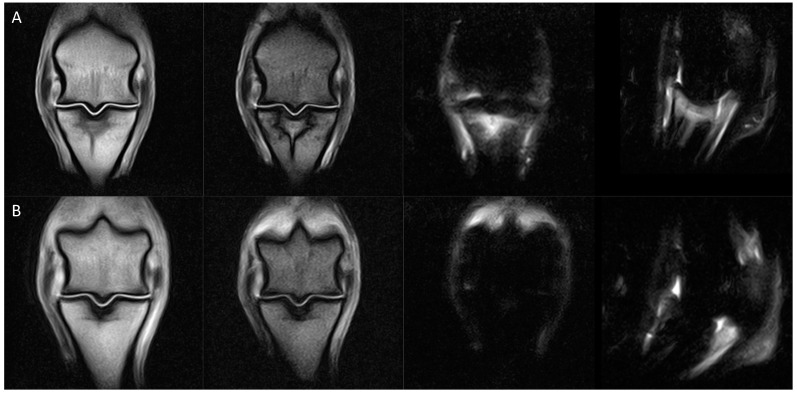
From left, dorsal T1W GRE, dorsal T2 GRE, dorsal STIR and sagittal STIR, images of the right hindlimb fetlock of a horse with static SGD MRI classification between (**A**) initial examination (classification 5) and (**B**) 3 months later during the rest period of the rehabilitation program (classification 5). Despite there being no change in classification, there are signs of healing as there is almost complete resolution of the bone oedema-like signal associated with the SG and the macrofissure line is only faintly appreciated. *Notes. T1 GRE, T1-weighed gradient recalled echo; T2 GRE, T2oW/, T2-weighted out of phase gradient recalled echo; STIR FSE, short tau inversion recovery fast spin echo*.

The study has identified shortcomings of this version of the SGD MRI classification system for longitudinal monitoring of SGD, and these should be improved before further use. Shortcomings include the lack of accounting for the extent of bone oedema-like signal in classifications 4a/b/c and 5 and subsequently a lack of accounting for decreases in extent of this pathological change. For example, even a marked reduction in the extent of bone oedema-like signal will not contribute to a change in classification, as it will only alter when there is complete resolution of bone oedema-like signal (Figure 6). In order to address some of these issues, the authors suggest that further division of classifications 4 and 5 may be necessary to account for the extent of bone oedema-like signal if it is shown to be associated with different outcomes in future studies. This could be incorporated using the following subgroups for bone oedema-like signal: (i) involving only the subchondral bone plate, (ii) reaching the level of the proximal physis/physeal scar and (iii) extending into the diaphysis. A similar limitation of the classification system is that fracture healing is shown to take the longest to occur proximally, in and/or adjacent to the proximal subchondral bone plate [15] and improvement in fissure size during the healing process will not be reflected by this classification system (Figure 6). Additionally, clarification of classification is required for cases where a hypointense line remains on all sequences at the site of a macrofissure but complete healing is suspected to have occurred.

In this study, performance has been used as an indicator of adequate management of SGD, as lameness tends to be intermittent in horses with SGD [2,5,7]. In the sample population, the proportion of horses recovering was relatively high, with 86% of horses returning to full work and competition after the initial rehabilitation period, the majority after conservative treatment. The three cases lost to follow-up were most likely retired or used for low-level competition or pleasure riding, as no competition results were found. Previous publications reveal marked variation in the percentages of horses with SGD that recover to perform at their intended use and level, with successful and unsuccessful performance outcomes described following both conservative and surgical treatments [2,3,4,5,7,13,15,16,17,19,20,21,22]. In horses diagnosed with SGD on MRI, high success rates of return to intended use were seen in thoroughbred racehorses [3,7] and, in a study of non-racing horses, 6/7 horses (85%) returned to full work after conservative treatment, with the recovery of the remaining horse complicated with a concurrent injury [5]. Conversely, another study of sports horses with SGD reported that only 9/17 horses (53%) returned to athletic function, with similar results for conservative and surgical treatment [4]. The poorest outcomes were reported in a study of warmblood horses, where 6/19 horses (31.5%) returned to their previous level of athletic use [2]. In other studies where short incomplete sagittal fractures were diagnosed in non-racehorses using radiography or CT rather than MRI, one study found better outcomes in those treated surgically than conservatively [13] and another found that 87% of horses that were treated surgically returned to previous activity levels [19]. It has been suggested that presence of osteoarthritis, cartilage damage and chronicity of pathology may contribute to poor outcomes [2]. These features are not incorporated in the current version of the SGD MRI classification system, and the effect of these factors should be evaluated further. Additionally, the significance of persistence of bone oedema-like signal on outcome is also unclear and needs further investigation. It has been proposed that hyperintense STIR signal associated with trauma typically resolves with rest, while pathologic changes associated with degenerative injury results in persistent high signal intensity and may result in chronic lameness [2,34].

In this study, the overall mean ± sd time to full recovery for those that returned to full work was 9.4 ± 4.4 months, which is similar to previously reported timings [3,19,20]. The range of time taken to return to full performance in the current study was between 5 and 20 months, a wide range which is also corroborated by the existing literature where recovery times as short as 4 months and as long as 26 months are reported [3,19,20]. Following optimisation and validation of the classification system, statistical comparison of the time taken to reach full performance for different classification groups should be investigated in future research projects with larger case numbers. Higher values for time to recovery may reflect an increased time needed to heal as detected by clinical responses to increasing workloads and findings on clinical examinations or follow-up radiography, or that rehabilitation programs are formulated with a preconception that more severe pathology will require a longer rehabilitation in mind. In future studies, the classification system may be investigated both with and without sub-classification to establish whether sub-classification is necessary.

Some horses did not undergo follow-up MRI examinations because they underwent immediate surgical treatment following the initial MRI diagnosis. MRI cannot reliably be used in limbs with metal implants, as the susceptibility artefacts cause large regions of signal void and distortion of the anatomy, therefore these horses did not form part of the study [35]. A comparison of the initial MRI findings between those which underwent immediate internal fixation and those which underwent conservative treatment would potentially uncover whether presence of certain MRI features was associated with certain treatment decisions. The details of subsequent rehabilitation programs and performance outcomes could also be compared to see if these differ significantly between these groups, but this was outside the scope of the article.

A limitation of the study is that decisions on the management of the horses were not standardised. Recommendations were made on a case-by-case basis, and choices would have been influenced by the clinical history, clinical findings, preferences of the referring veterinarians, owner preferences, financial considerations and/or radiologist’s advice. Investigation into the choice of management was outside the scope of the article, and data pertaining to these factors were highly variable and not available in many cases. With regards to the MRI findings, if there was bone oedema-like signal present on follow-up MRI, generally the radiologist would recommend further rest until resolution. If there was a decrease in bone oedema-like signal and then stabilisation to a mild degree over 2–3 sequential MRIs but without complete resolution, it was recommended that the horse could increase workload. In this situation, the decision would be made in conjunction with the referring veterinarian and with subsequent close clinical monitoring of the horse.

Only one horse was rescanned during an advanced phase of rehabilitation, with the initial classification of 4a maintained at 5 months. Although the horse was lame at the time of repeat MRI, this was attributed to other causes, and it did successfully re-enter competition 2 months later and was able to perform throughout the following 6 months to time of data collection. No other horses were scanned towards the end of their rehabilitation program, precluding evaluation of MRI findings in the period immediately before embarking on full work. Such information would improve understanding of lesion healing, and further research is needed in this area to determine whether there are characteristic features that increase the risk of recurrence of clinically apparent disease. Additionally, without a more consistent schedule of follow-up MRI examination, it is not possible to determine the proportion of horses in which complete resolution of bone oedema-like signal occurs and the time scale of this.

The duration of the rehabilitation periods for the horses that re-presented for MRI following a period in full work varied between 6 and 15 months, and the time spent in full work before re-presentation ranged from 2 to 15 months. In the horses completing the rehabilitation period in a quicker timing than average, it is suspected that an exacerbation of pathology occurred due to insufficient healing. In other cases, the trigger of the relapse is not identified. A limitation of the study is the absence of information on recurrence of clinically relevant SGD in the horses that did not re-present for MRI. In this instance, the horses may have received further MRI evaluation and/or treatment at another referral facility, undergone conservative treatment as guided by the primary veterinarian without further MRI or been retired.

Additional limitations of the study are its retrospective and descriptive design with inclusion based on diagnoses in original MRI reports. The degree of detail pertaining to clinical history and clinical examination was variable, and therefore lameness severity and localisation of pain causing lameness are not presented. However, it should be noted that the majority of MRI referrals at Equitom Equine Clinic come from veterinarians with an extensive experience in orthopaedics and sports medicine. Additionally, the effects of bilateral SGD were not investigated, and other causes of lameness were not taken into consideration, which may result in confounding bias.

The results of this study have highlighted that there are several aspects of the SGD MRI classification system that should be altered and optimised before further use. Furthermore, multi-modality comparisons should be performed to establish the accuracy of MRI in identifying lesions such as microfissures, as this may conclude that certain classification groups may be merged. Eventual development of a verified, condensed version is essential to ensure it could be used in a clinical setting. Despite the study being performed at a hospital that has a relatively large sports horse caseload with large number of MRI examinations, the sample sizes for classification groups are low, hindering statistical analysis. Following establishment of a robust classification system, a much larger prospective multicentre study with multiple observers, regimented MRI schedules and rehabilitation programs would be the optimum way to further investigate the progression of SGD on MRI images and associations with performance outcomes.

## 5. Conclusions

Variability in evolution of SGD on follow-up MRI was confirmed in this study. During the rest phase of the rehabilitation period, the SGD MRI classification worsened in some horses. Importantly, it was shown that classifications 4b (bone oedema-like signal with subchondral microfissure) and 4c (bone oedema-like signal with subchondral demineralisation) should be considered potential prodromal or imminent fissure pathology. Although the risk of complete fracture if kept in work cannot be directly evaluated, as the horses in the study were withdrawn from exercise, it is recommended that horses with SGD MRI classifications 4b, 4c and 5 (incomplete macrofissure/fracture) are carefully managed to minimise the risk of catastrophic fracture propagation [5,13]. In this study, successful return to full training and competition was achieved in a large proportion of horses with SGD, however, multiple horses re-presented due to recurrent lameness after varying time periods. Further investigation into patterns of progression of SGD over time is required. This would optimally occur in the form of a prospective multicentre study with different populations of horses and a regimented MRI examination schedule. The SGD MRI classification system used in this study needs to be verified and further optimised before large-scale studies are performed and simplified before it is used in clinical practice, however it is envisaged that a future derivative of the classification system may be used to better formulate prognoses, treatment plans and rehabilitation programs for horses with SGD.

## Figures and Tables

**Table 1:** Sagittal groove disease (SGD) magnetic resonance imaging (MRI) classification system.

SGD MRI Classification		Sub-Classifications and Description of Key Osseous Changes in the Sagittal Groove of the Proximal Phalanx	Absent Concurrent Features	Potential Concurrent Features
0	Normal	No abnormalities	N/A	N/A
1	Small subchondral defect	(a) Minor, shallow defect in the chondro-osseous junction (typically ≤1 mm depth ± visible in only one slice)	Demineralisation, osseous densification, bone oedema-like signal	N/A
		(b) Microfissure (proximodistally oriented, narrow, linear defect in the chondro-osseous junction that is contained within the subchondral bone plate, ≤3 mm length)		
2	Osseous densification	(a) Mild osseous densification of the subchondral ± trabecular bone, not extending to the proximal physis/physeal scar (proximodistal extent less than the equivalent depth of the SG)	Microfissure, demineralisation, bone oedema-like signal	Minor subchondral defect
		(b) Moderate to severe osseous densification of the subchondral ± trabecular bone, extending to or beyond the proximal physis/physeal scar (proximodistal distance greater than one times the depth of the SG)		
3	Subchondral microfissure with osseous densification	(a) Subchondral microfissure (≤3 mm length) with mild osseous densification (does not reach the proximal physis/physeal scar)	Subchondral demineralisation or bone oedema-like signal	
		(b) Subchondral microfissure (≤3 mm length) with moderate to severe osseous densification (extending to or beyond the proximal physis/physeal scar)		
4	Bone oedema-like signal within the subchondral ± trabecular bone	(a) Bone oedema-like signal within the subchondral ± trabecular bone	Microfissure, demineralisation	Minor subchondral defect, osseous densification of any extent
		(b) Bone oedema-like signal within the subchondral ± trabecular bone with microfissure (short proximodistally oriented, narrow, linear defect contained within the subchondral bone plate (≤3 mm))	Demineralisation	Osseous densification of any extent
		(c) Bone oedema-like signal within the subchondral ± trabecular bone with subchondral demineralisation (unipartite or tripartite regions of demineralisation/resorption within the subchondral bone plate)		Microfissure, osseous densification of any extent
5	Incomplete macrofissure/fracture	Proximodistally oriented linear signal abnormality (>3 mm length; unipartite or tripartite configuration) extending through the subchondral bone and terminating within the trabecular bone	N/A	Demineralisation, osseous densification or bone oedema-like signal of any extent
6	Complete fracture	Proximodistally oriented linear signal abnormality extending through the subchondral and trabecular bone and exiting at the diaphyseal cortex or distal subchondral bone plate with the creation of two or more fragments	N/A	Demineralisation, osseous densification or bone oedema-like signal of any extent

Footnotes. Osseous densification represented by abnormal low signal intensity of the subchondral and/or trabecular bone on T1 GRE and T2 FSE. Bone oedema-like signal represented by T1 GRE hypointensity, STIR FSE hyperintensity and intermediate or increased intensity on T2 GRE with rim of hypointensity (fat water cancellation phenomenon). Demineralisation represented by regions within the subchondral bone plate, which are hyperintense on all sequences. Unipartite describes a singular lesion. Tripartite describes a configuration of three parallel dorsopalmarly/plantarly (± proximodistally) oriented lines, typically two hyperintense lines flanking a hypointense line, similar to the “chromosome-like” appearance described on CT [1]). *Notes*. *N/A, none applicable*; T1 GRE, T1-weighed gradient recalled echo; T2 FSE, T2-weighted fast spin echo; STIR FSE, short tau inversion recovery fast spin echo; T2 GRE, T2-weighted out of phase gradient recalled echo (T2*oW).

**Table 2:** The number of MRI examinations, presence of lameness and recent performance/rehabilitation phase with respect to time since initial MRI examination for 29 horses with sagittal groove disease (32 limbs) from the initial examination and during the initial rehabilitation period. Two horses (three limbs) did not undergo follow-up scan during the initial rehabilitation period, only in a subsequent period.

		Time Since Initial MRI Examination (Months)						
		0	0–3	3–6	6–9	9–12	12–18	15–18
Total number of limbs examined (n)		32	11	22	3	1	0	1
Lameness	Lame (n)	31	2	2	0	0	0	0
	Sound (n)	1	6	16	3	0	0	0
	N/R (n)	0	3	4	0	1	0	1
Recent performance/rehabilitation phase	Rest (n)	2	11	15	0	1	0	1
	Base rehab (n)	0	0	6	3	0	0	0
	Advanced rehab (n)	0	0	1	0	0	0	0
	Full (n)	30	0	0	0	0	0	0

Footnotes. Initial MRI examination at T = 0; N/R—no record or not examined; Rest—box rest, paddock rest, hand/ridden walking; Base rehab—rehabilitation program with flat work, basic movements and cavaletti only; Advanced rehab—rehabilitation program including advanced movements and jumping; Full—normal level of training/competition.

**Table 3:** Sagittal groove disease (SGD) MRI classification, duration of rehabilitation periods, time to reach full performance and performance levels for the horses that re-presented for MRI examination due to recurrence of lameness.

	Initial Period			Subsequent Period/s				
Horse	SGD MRI Classification/s	Time to Resume Full Training/Competition (Months)	Level of Performance Compared with Previously	Time in Full Work (Months)	Time since Initial MRI of the Initial/Previous Period	SGD MRI Classification/s ^¶^	Time to Resume Full Training/Competition (Months)	Level of Performance Compared with Previously
1	4a (RF)	9	Same	11	20	4a (RF) 4a (LF) ^†^	14	Lower
2	4b (RF)	6	Same	15	21	5/4b (RF)	11	Unknown
3	5 (RH)	>9	Same	Unknown	24	4c/4a (RH)	Ongoing rehabilitation ^‡^	-
4 (A)	4b/5 (LF) 2a/4a (RF)	8	Unknown	8	16	4c/5 (LF)	8 ^§^	Unknown
5 (C)	4a (LF) 4b (RF)	Unknown	Unknown	Unknown	39	4c (LF) 5 (RF)	Unknown	Unknown
6 (D) ^	4a (RF)	15	Higher	3	18	4a/5 (RF)	6	Lower
	-	-	-	2	8	4a (RF) 4a/4a (LF)	12	Lower

Footnotes. Horse numbers 1–6 correspond to Figure 3; letters A, C and D correspond to Figure 2 and Figure 3 and indicate bilateral forelimbs of the same horse; ^¶^, in the case of multiple examinations being performed during the rehabilitation period these are separated by a forward slash; ^†^, LF not shown on the graph in Figure 3 as only had one MRI examination; ^‡^, horse currently 5 months into rehabilitation program, aiming to reach full performance in a further 3 months; ^§^, CT-guided cortical screw fixation of the PP performed on the LF after 4 months of rehabilitation, performing after an additional 4 months; ^, horse re-presented for MRI due to recurrent lameness on two occasions after undergoing two separate rehabilitation periods followed by re-entry into full work.

## Data Availability

Data available on request due to restrictions; the data presented in this study may be available on request from Z.J. and T.M. with the permission of Equitom Equine Clinic. The data are not publicly available due to patient/client confidentiality. The research data are allowed to be used and published.

## Supplementary Materials

The following supporting information can be downloaded at: https://www.mdpi.com/article/10.3390/ani14010034/s1, Supplementary File S1: Fetlock MRI acquisition protocols used in 33 limbs diagnosed with sagittal groove disease of the proximal phalanx; Supplementary File S2: Visual guide to MRI semi-quantitative grading scheme for sagittal groove disease of the proximal phalanx. Reference [1] are cited in the supplementary materials.
