# Supplementary material for: Follow-Up Magnetic Resonance Imaging of Sagittal Groove Disease of the Equine Proximal Phalanx Using a Classification System in 29 Non-Racing Sports Horses
Source: Animals (Basel). 2023 Dec 21;14(1):34. doi: 10.3390/ani14010034 (PMC10778323; doi:10.3390/ani14010034)
Supplement: Supplementary file 1 [file animals-14-00034-s001.zip › Supplementary file S1.pdf]

Supplementary file S1:
Fetlock MRI acquisition protocols used in 33 limbs diagnosed with sagittal groove disease (SGD) of the proximal phalanx (PP)

## Materials and methods

The typical acquisition protocol for a fetlock with SGD using an open 0.27T permanent magnetic resonance system (Hallmarq EQ2, Guildford, UK) would include:

- T1 GRE/ T2 GRE / STIR FSE FRO oriented on the PP
- T1 GRE/ STIR FSE SAG
- T1 GRE/ T2 FSE/ T2 GRE / STIR FSE TRA oriented on the condyle, with additional sequences oriented on the PP if lesion extent not clear.
- All slice thickness 5mm, with additional 'HR FAST' with slice thickness 3,5mm if lesion not detailed enough.
- Motion correction techniques selected based on the level of cooperation of the patients. FAST (2 experiments) attempted first, sometimes SFAST (1 experiment) if too much motion.

## Results

Figure: Bar chart showing MRI sequences performed during sequential examination of 33 fetlocks with sagittal groove disease, displayed as percentages of limbs per exam

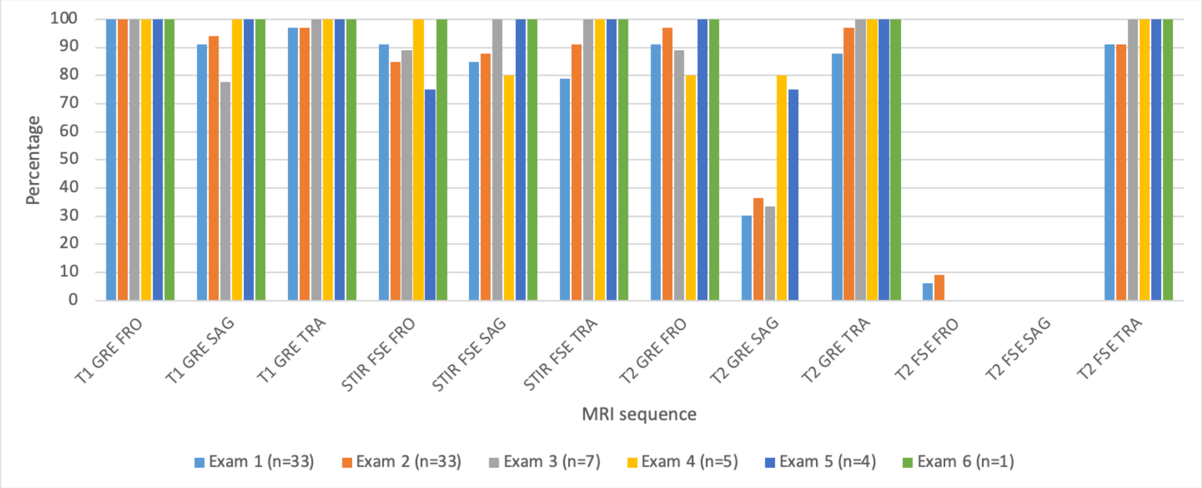


*Notes*

*T1 GRE, T1-weighed gradient recalled echo ; STIR FSE, short tau inversion recovery fast spin echo; T2 GRE, T2-weighted out of phase gradient recalled echo (T2*oW); T2 FSE,T2-weighted fast spin echo; FRO, dorsal ('frontal') plane; SAG, sagittal plane; TRA, transverse plane.*
