# Supplementary material for: Follow-Up Magnetic Resonance Imaging of Sagittal Groove Disease of the Equine Proximal Phalanx Using a Classification System in 29 Non-Racing Sports Horses
Source: Animals (Basel). 2023 Dec 21;14(1):34. doi: 10.3390/ani14010034 (PMC10778323; doi:10.3390/ani14010034)
Supplement: Supplementary file 1 [file animals-14-00034-s001.zip › Supplementary file S2.pdf]

Supplementary file S2:
Visual guide to MRI semi-quantitative grading scheme for sagittal groove disease of the proximal phalanx

Proximal phalanx: sagittal groove

3 subregions - dorsal, middle and palmar/plantar thirds

Locations categorised as lateral sloping parasagittal zone, mid-sagittal zone and medial sloping parasagittal zone

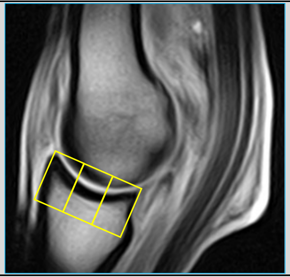

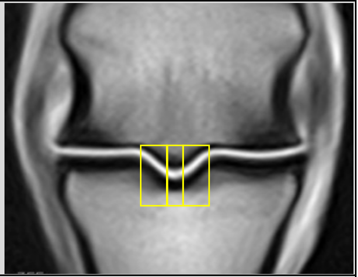

Criteria	Description	Grading				
Subchondral bone defects	An interruption of the subchondral bone	0 = Absent<br><br>Smooth and regular chondro-osseous junction	1 = Minor indentation or irregularity of the chondro-osseous junction<br><br>Shallow and/or visible in one slice	2 = Microfissure (≤ 3mm length)<br><br>Proximodistally-oriented, narrow, linearity extending from the chondro-osseous junction and not exiting the distal aspect of the subchondral bone plate	3 = Short macrofissure (>3mm and <15mm length)<br><br>Proximodistally-oriented, narrow, linear defect extending through the (thickened) subchondral bone plate and trabecular bone	4 = Long macrofissure/ fracture (≥15mm length)<br><br>Proximodistally-oriented, narrow, linear defect extending through the (thickened) subchondral bone plate and trabecular bone
		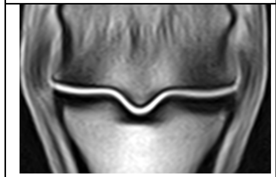	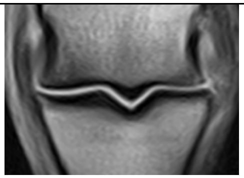	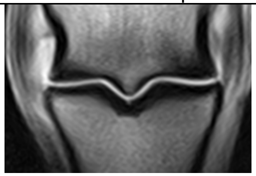	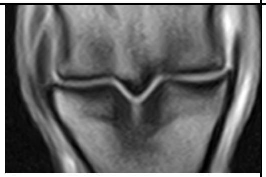	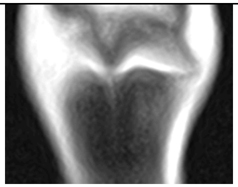
	Location categorised as: Mid-sagittal with proximodistal orientation, originating at the lateral margin of the mid-sagittal zone and/or with a distolateral oblique component, originating at the medial margin of the mid-sagittal zone and/or with a distomedial oblique component, lateral parasagittal, medial parasagittal.<br><br>Configuration categorised as: Unipartite (singular lesion), tripartite (three parallel dorsopalmarly/plantarly ± proximodistally oriented lines, typically two hyperintense lines flanking a hypointense line, similar to the "chromosome-like" appearance as described on CT [1]).<br><br>Examples shown (L to R) of mid-sagittal (unipartite), mid-sagittal (tripartite), oblique component.			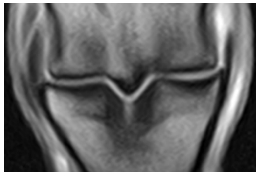	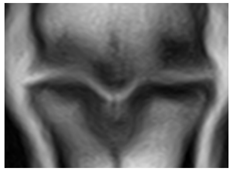	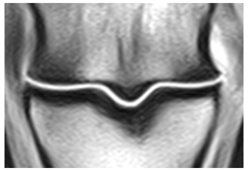
	Size measured (proximodistal x dorsopalmar/plantar x mediolateral) (mm) on T1 GRE sequences where possible, values <1mm entered as 1mm					

Subchondral demineralisation	Regions within the subchondral bone plate which are hyperintense on all sequences	0 = Absent<br>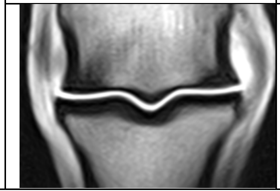	1 = Unipartite<br><br>Single site<br>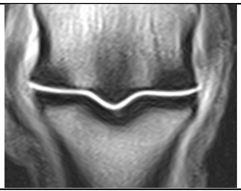	2 = Tripartite/ “chromosome-like” [1]<br><br>Two hyperintensities separated by hypointensity<br>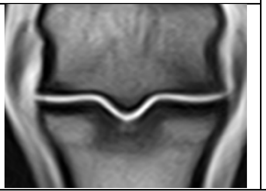	* Suspected osseous cyst-like lesions included in category and noted<br><br>Well defined osseous defect with sclerotic rim, suspected fluid or gelatinous content	* Proximodistally-oriented hyperintense linearities not counted as demineralisation<br>(-> categorised as fissure – grade 2, 3 or 4 subchondral bone defects, see above)
	Location recorded as maximal point: mid-sagittal, medial or lateral parasagittal					
	Size of demineralised region (proximodistal x dorsopalmar/plantar x mediolateral)(mm) measured on T1 GRE sequences					
Osseous densification	Abnormal low signal intensity of the subchondral and/or trabecular bone on T1 GRE and T2 FSE<br><br>Extent visually estimated using proximodistal distance from articular surface	0 = Normal subchondral bone plate thickness and transition to trabecular bone (± ≤ 3mm)<br>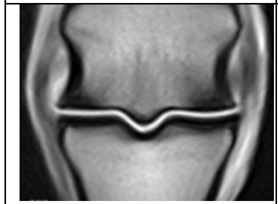	1 = Hypointense signal increased, distance less than the depth of the sagittal groove<br>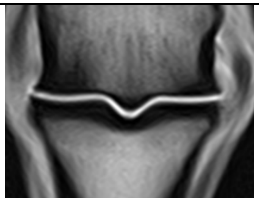	2 = Hypointense signal increased, distance greater than one times the depth of the sagittal groove and extending no further than the physis/physeal scar<br>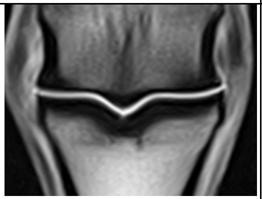	3 = Hypointense signal increased, extending further distally than the physis/ physeal scar and into the diaphysis<br>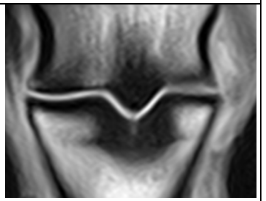	
	Location of the densification recorded as centred: mid-sagittally (biaxially symmetrical), medially or laterally					

Bone oedema-like signal	T1 GRE hypointensity, STIR FSE hyperintensity, intermediate or increased intensity on T2 GRE (T2*oW) with rim of hypointensity (fat water cancellation phenomenon, FWC)<br><br>Extent visually estimated using proximodistal distance from articular surface	0 = Absence of relevant bone oedema-like signal<br><br>No clear STIR hyperintensity; T2* hyperintense foci or thin line within the subchondal bone plate which parallel the anatomical contour of the articular surface are not considered relevant and therefore may be present	1 = Abnormal signal involving only the subchondral bone plate.<br><br>STIR hyperintensity may be present (not always visualised due to artefacts or concurrent osseous densification); larger or thicker region of hyperintense or intermediate T2* signal, with clear rim of hypointense FWC	2 = Abnormal signal involving the subchondral bone plate and extending into the trabecular bone, up to the level of the proximal physis/ physeal scar	3 = Abnormal signal involving the subchondral bone plate and extending into the diaphyseal trabecular bone	4 = Abnormal signal extending into the diaphyseal trabecular bone, with distally directed linear extension
		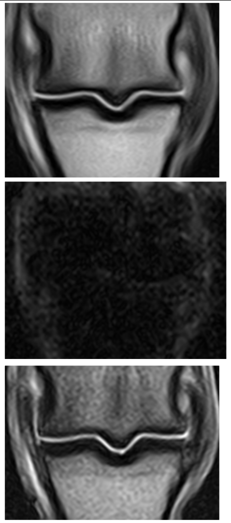	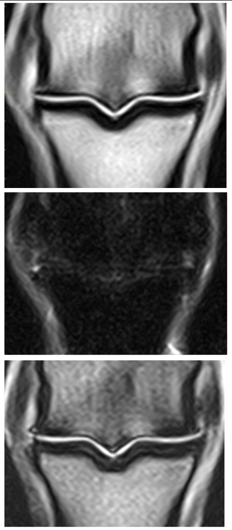	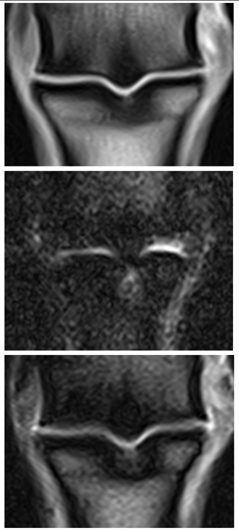	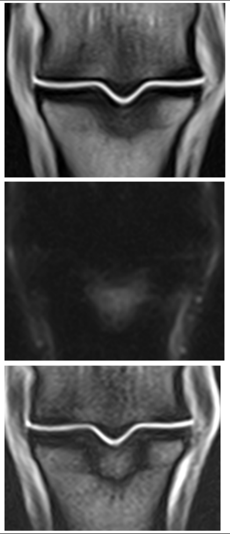	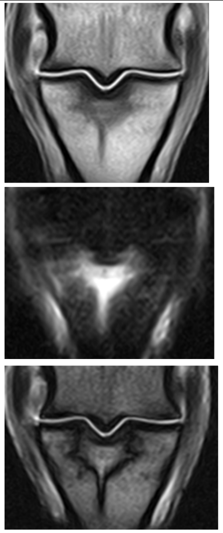
	Location of bone oedema-like signal recorded as being centred mid-sagittally, medially or laterally					

References
1. Brünisholz, H.P.; Hagen, R.; Fürst, A.E.; Kuemmerle, J.M. Radiographic and Computed Tomographic Configuration of Incomplete Proximal Fractures of the Proximal Phalanx in Horses Not Used for Racing. Veterinary Surgery 2015, 44, 809–815, doi:10.1111/vsu.12364.
